# The Quantitative Relationship among the Number of the Pacing Cells Required, the Dimension, and the Diffusion Coefficient

**DOI:** 10.1155/2020/3608015

**Published:** 2020-06-25

**Authors:** Yue Zhang, Guisheng Yin, Kuanquan Wang

**Affiliations:** ^1^College of Computer Science and Technology, Harbin Engineering University, Harbin 150001, China; ^2^School of Computer Science and Engineering, University of New South Wales, Sydney, NSW 2052, Australia; ^3^School of Computer Science and Technology, Harbin Institute of Technology, Harbin 150001, China

## Abstract

The purpose of the paper is to derive a formula to describe the quantitative relationship among the number of the pacing cells required (NPR), the dimension *i*, and the diffusion coefficient *D* (electrical coupling or gap junction *G*). The relationship between NPR and *G* has been investigated in different dimensions, respectively. That is, for each fixed *i*, there is a formula to describe the relationship between NPR and *G*; and three formulas are required for the three dimensions. However, there is not a universal expression to describe the relationship among NPR, *G*, and *i* together. In the manuscript, surveying and investigating the basic law among the existed data, we speculate the preliminary formula of the relationship among the NPR, *i*, and *G*; and then, employing the cftool in MATLAB, the explicit formulas are derived for different cases. In addition, the goodness of fit (*R*^2^) is computed to evaluate the fitting of the formulas. Moreover, the 1D and 2D ventricular tissue models containing biological pacemakers are developed to derive more data to validate the formula. The results suggest that the relationship among the NPR, *i*, and the *G* (*D*) could be described by a universal formula, where the NPR scales with the *i* (the dimension) power of the product of the square root of *G* (*D*) and a constant *b* which is dependent on the strength of the pacing cells and so on.

## 1. Introduction

In the normal heart, the electrical pulses are initiated in the genuine pacemaker–sinoatrial node (SAN), which generates the excitation automatically [1, 2]. However, the dysfunction of the SAN would lead to a variety of manifestations, including fibrillation, arrhythmias, and heart failure [3–5]. To treat these diseases, the best way at present is to implant electronic devices [6]. There are more than 200,000 patients suffering electrical pacemaker implantation every year in USA alone [7]. Nevertheless, the limitations of the devices could not be ignored, including lead malfunction, infection, short battery lifespan, and thrombosis and so on [8–12].

As a consequence, the biological pacemaker has been attracting the attention of the researchers to overcome the disadvantages of the electronic devices [13–16]. One of the popular strategies is to create biological pacemakers in the ventricle [17–19], because the thicker ventricular wall is more conductive to the biological experiments.

For a successful pacemaker, an important feature is the source-to-sink match. The pacemaker acts as the source to drive the adjacent resting cardiac tissue which is considered as the sink. The sink is at a resting state until it is stimulated by the pulses from the source. That is, the source must be strong enough to drive the sink to break the threshold to depolarize. The depolarization of the sink would fail if the source-to-sink mismatch is too large, leading to the failure of the excitation propagation. The topic has been investigated extensively in the SAN [20, 21]. The research demonstrates that the gap junction (electrical coupling) plays an important role in the source-to-sink mismatch [22–24]. The coupling conductance is much weaker in the SAN than that in other cardiac tissues [25, 26]. The poor coupling reduces the suppression from the adjacent hyperpolarized tissue, shielding the depolarization of the pacing tissue to overcome the source-to-sink mismatch [27].

The ability of the source (the pacing tissue) to drive the sink (the cardiac tissue) depends on the number of the pacing cells, the gap junction conductance, and the strength of the pacing cells. For a successful pacing and driving, what is the relationship between the NPR and the gap junction conductance? Tveito et al. showed that the NPR was proportional to the square root of the gap junction conductance in the1D strand and to the gap junction conductance in the 2D tissue [28, 29]. Xie et al. validated the consistent conclusion for the propagation of the action potential triggered by early (EAD) and delayed afterdepolarizations (DAD), and they further concluded that the NPR scaled with the 1.5 power of the gap junction conductance in the 3D volume [30].

The analysis of the relationship between the NPR and the gap junction conductance to overcome the source-to-sink mismatch has been performed in different dimensions, respectively. That is, for each fixed dimension *i*, there is a formula to describe the relationship between NPR and the gap junction *G*, and three formulas are required for all the three dimensions. However, to our knowledge, there is not a universal expression to show the precise relationship among the NPR, gap junction *G*, and dimension *i* together. In the manuscript, using the computational model to obtain the simulation data, and together with the data from reference [30], we set out to further probe the topic and try to address a universal formula to show how many pacing cells in different dimensions are required to overcome the source-to-sink mismatch to make the pulses propagate from the pacing tissue with variable gap junction conductance into the surrounding regions.

Firstly, surveying and analyzing the existing data [30] on the whole, we speculate and derive a basic format of the formula describing the relationship among the NPR, *i*, and *G*. And then, the accurate formulas were calculated, validated, and evaluated for different cases. Thirdly, based on the TNNP06 ventricular single-cell model [31], the pacing cell model is addressed by depressing *I*_K1_ to 0.05 nS/pF. Next, using the reaction diffusion equation, the 1D and 2D models are developed by coupling the pacing cells and ventricular myocytes together. From the models, the NPRs are obtained corresponding to variable gap junction conductance in different dimensions. Finally, the simulation data are utilized to verify the formula derived previously.

## 2. Data and Methods

In this section, we introduce the models of the single cell and the tissue from which the simulation data are derived; and then, the related data from other reference are listed.

### 2.1. The Model of the Single Cell

For the single cell, we employ the well-established TNNP 2006 model of the human ventricular cells [31], which is shown in Equation (1).

(1) $$\begin{array}{c} \frac{dV}{dt}=-\frac{I_{\text{ion}}+I_{\text{stim}}}{C_{\text{m}}}, \end{array}$$

where

(2) $$\begin{array}{c} I_{\text{ion}}=I_{\text{Na}}+I_{\text{K}1}+I_{\text{to}}+I_{\text{Kr}}+I_{\text{Ks}}+I_{\text{CaL}}+I_{\text{NaK}}+I_{\text{NaCa}}+I_{\text{pK}}+I_{\text{pCa}}+I_{\text{bCa}}+I_{\text{bNa}}. \end{array}$$

In Equation (1), *V* is the transmembrane potential; *I*_ion_ is the sum of all the transmembrane ion currents; *I*_stim_ is the external stimulus current and is set to 0 in the study; *C*_m_ is membrane capacitance per unit surface area; *I*_K1_ is the inward-rectifier K^+^ current; *I*_Ks_, *I*_Kr_, and *I*_to_ are the outward slow, rapid, and transient and rectifier potassium currents, respectively.

In the simulation, *I*_K1_ is depressed to 0.05 nS/pF to obtain the robust pacing cells.

### 2.2. The Models of the 1D and 2D Tissues

**Figure 1:**
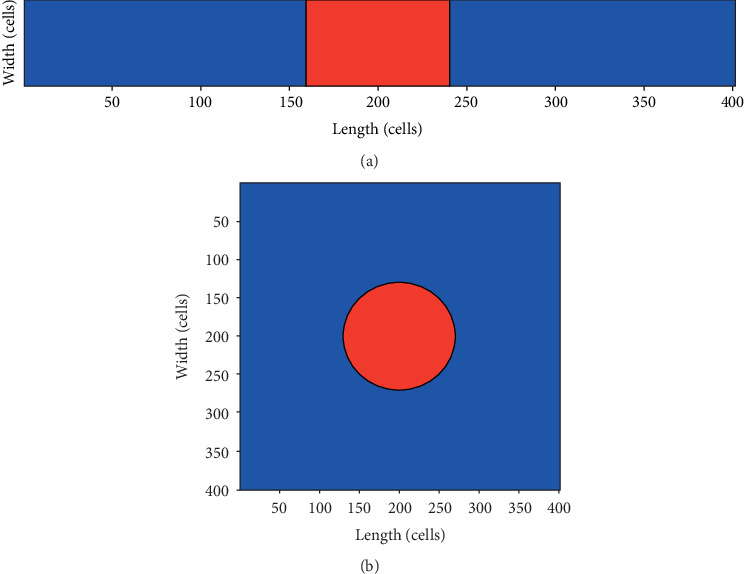
The settings of the tissues. The red area: the pacemaker; the blue region: the ventricular tissue. (a) The 1D strand. (b) The 2D tissue.

The settings of the 1D and 2D tissue are as shown in Figures 1(a) and 1(b), respectively. The central part, consisting of pacing cells, is the pacemaker, and the surrounding blue region is the ventricular tissue which is made up of endomyocardial myocytes.

The reaction-diffusion equation [31] is applied to describe the propagation of the electrical excitation wave in the 1D and 2D ventricular tissues, which is shown in Equation (3):

(3) $$\begin{array}{c} C_{m}\frac{\partial V}{\partial t}=-\left(I_{\text{ion}}+I_{\text{stim}}\right)+D\Delta V, \end{array}$$

where *D* is the diffusion coefficient with the normal value 15.4 mm^2^/s, which is corresponding to the electrical coupling (gap junction) in the tissue and describes the conductivity of the excitation; *∆* is the Laplace operator; and the other parameters are the same as those in Equation (1). In the simulation, the time step is set to 0.02 ms, and the space step is 0.15 mm.

### 2.3. The Data from the Existing Reference

Xie et al. investigated how the source overcame the mismatch to trigger the successful pulse propagation in different conditions [30]. The numbers of contiguous automatic cells required are listed in Tables 1 and 2 for various sources with diverse gap junctions in different dimensions.

In Tables 1 and 2, the abbreviations are listed in Table 3.

“EAD/normal,” “EAD/RRR,” “DAD/normal,” and “DAD/HF” are four pacemaker-tissue designs (cases).

### 2.4. The MATLAB Tool

The cftool in MATLAB is adopted for the fitting in the manuscript.

## 3. Results and Discussion

In this section, our aim is to derive a formula to fit the relationship among the NPR, the electrical coupling *D* (gap junction *G*), and the dimension *i*. And then, the accuracy of the formula is evaluated.

### 3.1. The Fitting Formula

From the observation and computation, we detect that in Tables 1 and 2, for each column of data, *N*_*i*_ (*i* = 1, 2, 3) is approximately a geometric sequence. The details are shown in Tables 4 and 5.

For all the 8 columns of data in Tables 4 and 5, *N*_2_/*N*_1_ is close to *N*_3_/*N*_2_, which infers that for a fixed *G*, *N*_*i*_ (*i* = 1, 2, 3) might be geometric sequences corresponding to *i*, and *N*_*i*+1_/*N*_*i*_ is the common ratio *q*(*G*). As a consequence, the expression of *N*_*i*_ of *i* can be described as

(4) $$\begin{array}{c} N\left(i,G\right)=m\left(G\right)*{\left(q\left(G\right)\right)}^{i} \end{array}$$

where *m*(*G*) and *q*(*G*) are functions of *G*, and *i* is the dimension.

What is more, the average values of *q*(*G*) for each *G* in different cases are computed according to the values of *N*_*i*+1_/*N*_*i*_ in Tables 4 and 5, respectively, which are listed in Table 6.

From Table 6, for all the cases “EAD/normal,” “EAD/RRR,” “DAD/normal,” and “DAD/HF,” it is found that ${\bar{q}}_{780}/{\bar{q}}_{125}$ is close to $\sqrt{G_{780}}/\sqrt{G_{125}}$, that is, *q* is proportional to $\sqrt{G}$. As a consequence, we set $q\left(G\right)=b*\sqrt{G}$, where *b* is a constant.

Therefore, the expression of *N*_*i*_ about *i* can be rewritten as Equation (5).

(5) $$\begin{array}{c} N\left(i,G\right)=m\left(G\right)*{\left(q\left(G\right)\right)}^{i}=m\left(G\right)*{\left(b\sqrt{G}\right)}^{i}=m\left(G\right)*b^{i}*{\left(\sqrt{G}\right)}^{i} \end{array}$$

According to [28–30], for a fixed *i*, *N*_*i*_ is proportional to the *i* power of the square root of the gap junction (coupling conductance) in the *i* dimensional tissue, and therefore, *N*_*i*_ could also be expressed as follows:

(6) $$\begin{array}{c} N\left(i,G\right)=f\left(i\right)*{\left(\sqrt{G}\right)}^{i}, \end{array}$$

where *f*(*i*) is a function of *i*, and *G* is the gap junction (coupling) conductance.

Considering Equations (5) and (6) together, for ∀*G* and ∀*i*, there is

(7) $$\begin{array}{c} f\left(i\right)=b^{i}*m\left(G\right). \end{array}$$

Therefore, *m*(*G*) must be a constant, set as *a*. And the formula of *N*(*i*, *G*) could be described as Equation (8).

(8) $$\begin{array}{c} N\left(i,G\right)=a*{\left(b\sqrt{G}\right)}^{i}, \end{array}$$

where *a* and *b* are the undetermined coefficients.

In the previous studies [28–30], only the relationship between NPR and *G* for each fixed dimension *i* is found. As a consequence, there are different formulas for different dimensions. That is, there are three formulas for all the three dimensions and three parameters should be determined. In the study, the relationship between NPR and *i* for each fixed *G* is also discussed. Based on the two relationships, Equation (8) is obtained. And only one formula is required for all the three dimensions and only two parameters are needed.

Using the cftool in MATLAB, we set out to fit the formula for the case “EAD/normal” according to the first two columns of data in Table 1. Knowing the format of the formula in Equation (8) in advance, we derive the formula for the case “EAD/normal”:

(9) $$\begin{array}{c} N\left(i,G\right)=0.6885*{\left(3.596\sqrt{G}\right)}^{i}. \end{array}$$

The computing *N*(*i*, *G*) for the case “EAD/normal” according to Equation (9) are shown in Table 7, compared with the original values.

The comparison in Table 7 illustrates that all the computing results are close to the corresponding original values; that is, the function *N*(*i*, *G*) fits the original data well.

**Figure 2:**
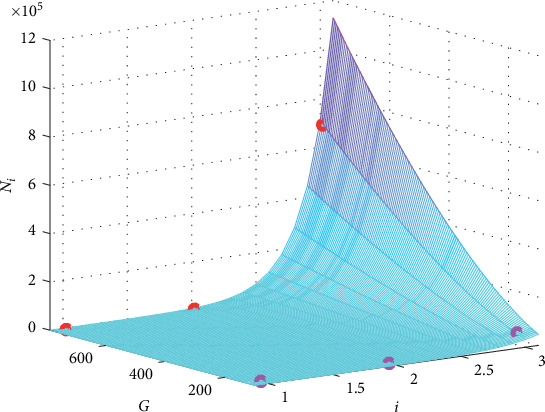
The graph of the fitting formula for the case “EAD/normal.” The red circles present the original *N*_*i*_ for *G*_gap_ = 780 nS, and the pink ones are for *G*_gap_ = 125 nS.

The graph of the fitting function is presented in Figure 2 together with the 6 original points in the first two columns of the data in Table 1.

From Figure 2, it could be seen intuitively that the small circles (the original values) cling to the surface of the function of *N*(*i*, *G*), which means that formula (9) is a good fitting.

The analogous operations are done for the cases “EAD/RRR,” “DAD/normal,” and “DAD/HF.” The formula for case “EAD/RRR” is shown in Equation (10).

(10) $$\begin{array}{c} N\left(i,G\right)=0.6308*{\left(2.558\sqrt{G}\right)}^{i}. \end{array}$$

**Figure 3:**
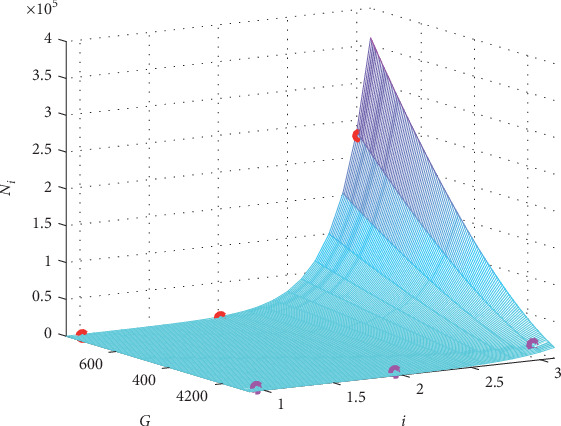
The graph of the fitting formula for the case “EAD/RRR”. The red circles present the original *N*_*i*_ for *G*_gap_ = 780 nS, and the pink ones are for *G*_gap_ = 125 nS.

And the graph of function (10) is presented in Figure 3 together with the original values of the case “EAD/RRR” in the second two columns of data in Table 1.

The formula for case “DAD/normal” is shown in Equation (11).

(11) $$\begin{array}{c} N\left(i,G\right)=0.7246*{\left(3.728\sqrt{G}\right)}^{i}. \end{array}$$

**Figure 4:**
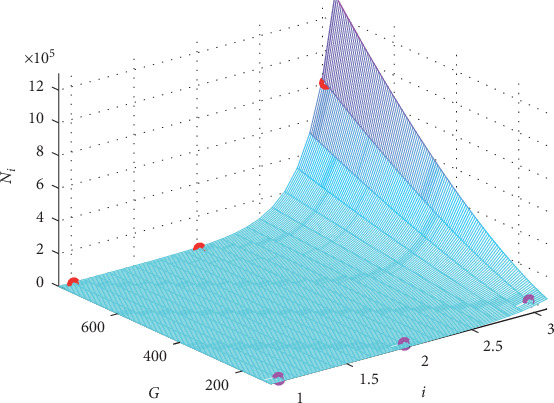
The graph of the fitting formula for the case “DAD/normal.” The red circles present the original *N*_*i*_ for *G*_gap_ = 780 nS, and the pink ones are for *G*_gap_ = 125 nS.

And the graph of function (11) is presented in Figure 4 together with the original values of case “DAD/normal” in the first two columns of data in Table 2.

The formula for case “DAD/HF” is shown in Equation (12).

(12) $$\begin{array}{c} N\left(i,G\right)=0.4126*{\left(1.784\sqrt{G}\right)}^{i}. \end{array}$$

**Figure 5:**
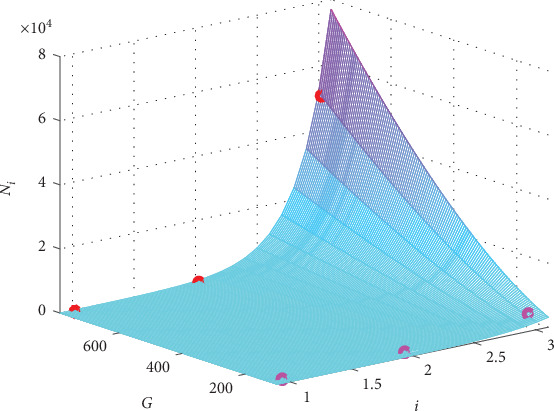
The graph of the fitting formula for the case “DAD/HF.” The red circles present the original *N*_*i*_ for *G*_gap_ = 780 nS, and the pink ones are for *G*_gap_ = 125 nS.

And the graph of function (12) is presented in Figure 5 together with the original values of case “DAD/HF” in the second two columns of data in Table 2.

### 3.2. The Evaluation of the Formula

In the previous section, we show the intuitive fitting results in Figures 2–5, which demonstrate that the formula in Equation (8) fits all the 4 cases accurately.

For a fixed *G*, the formula is an exponential function of *i*, and it could be transformed logarithmically to derive a linear function. And then, the goodness of fit (*R*^2^), which is an excellent tool to evaluate the linear function, is calculated to evaluate the fitting.

Taking log of both sides of Equation (8), we can obtain

(13) $$\begin{array}{c} y=\ln\left(N\left(i,G\right)\right)=\ln a+\left(\ln b+\ln\sqrt{G}\right)*i. \end{array}$$

And the linear expression for the case “EAD/normal” is

(14) $$\begin{array}{c} y=\ln\left(N\left(i,G\right)\right)=\left(1.2798+\ln\sqrt{G}\right)*i‐0.3732. \end{array}$$

**Figure 6:**
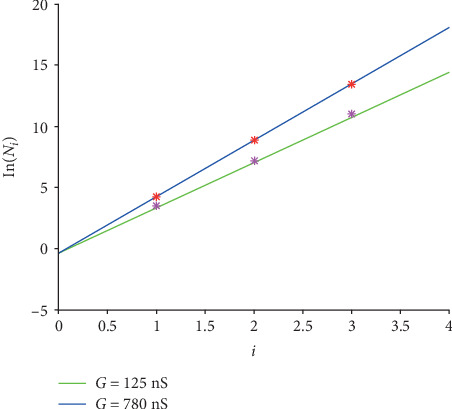
The linear graphs of function (14) for the case “EAD/normal.” The green line is for *G* = 125 nS and the blue one is for *G* = 780 nS. And the pink stars are the logarithmic original *N*_*i*_ for *G* = 125 nS, and the red ones are for *G* = 780 nS.

The graphs of function (14) for *G* = 125 nS and *G* = 780 nS are shown in Figure 6. And the 6 logarithmic original *N*_*i*_ in Table 1 for case “EAD/normal” are drawn in pink for *G* = 125 nS and in red for *G* = 780 nS, respectively.

Figure 6 illustrates that the formula fits the original values well intuitively. Furthermore, *R*^2^ is computed to evaluate the fitting quantitatively.

The closer *R*^2^ is to 1, the better the fitting is. And *R*^2^ values are 0.9964 and 1.0000 for *G* = 125 nS and for *G* = 780 nS, respectively. That is, the fitting for case “EAD/normal” is accurate and acceptable.

**Figure 7:**
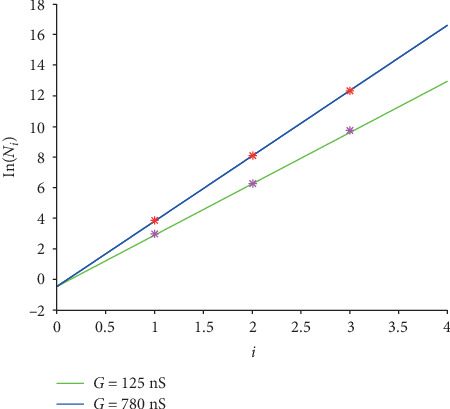
The linear graphs of function (15) for case “EAD/RRR.” The green line is for*G* = 125 nS and the blue one is for *G* = 780 nS. And the pink stars are the logarithmic original *N*_*i*_ for *G* = 125 nS, and the red ones are for *G* = 780 nS.

**Figure 8:**
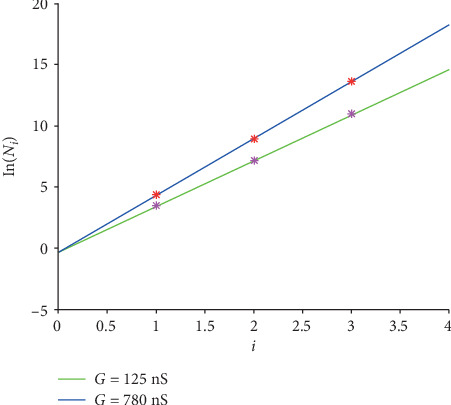
The linear graphs of function (16) for case “DAD/normal.” The green line is for *G* = 125 nS and the blue one is for *G* = 780 nS. And the pink stars are the logarithmic original *N*_*i*_ for *G* = 125 nS, and the red ones are for *G* = 780 nS.

**Figure 9:**
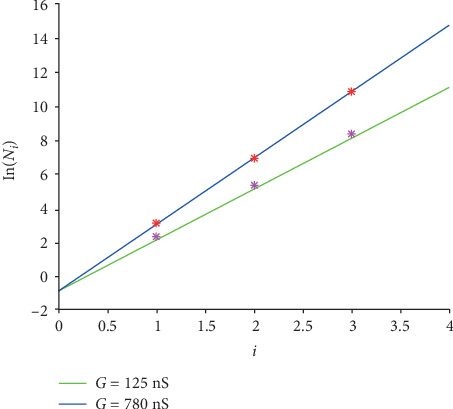
The linear graphs of function (17) for case “DAD/HF.” The green line is for *G* = 125 nS and the blue one is for *G* = 780 nS. And the pink stars are the logarithmic original *N*_*i*_ for *G* = 125 nS, and the red ones are for *G* = 780 nS.

The linear transformation for cases “EAD/RRR,” “DAD/normal,” and “DAD/HF” is expressed in Equations (15), (16), and (17), and the corresponding graphs are shown in Figures 7–9.

(15) $$\begin{array}{c} y=\ln\left(N\left(i,G\right)\right)=\left(0.9392+\ln\sqrt{G}\right)*i‐0.4608, \end{array}$$

(16) $$\begin{array}{c} y=\ln\left(N\left(i,G\right)\right)=\left(1.3159+\ln\sqrt{G}\right)*i‐0.3221, \end{array}$$

(17) $$\begin{array}{c} y=\ln\left(N\left(i,G\right)\right)=\left(0.5789+\ln\sqrt{G}\right)*i‐0.8853. \end{array}$$

The goodness of fit (*R*^2^) for all the four cases are listed in Table 8.

Intuitively, all the lines in Figures 7–9 fit the corresponding original values closely. Moreover, all the *R*^2^ values are no less than 0.99, inferring that the fitting are accurate.

### 3.3. The Verification of the Formula

In this section, using the models in Section 2.1 and Section 2.2, we simulate the biological pacemakers with different couplings to derive the corresponding number of the pacing cells required. And then, we set out to verify whether formula (8) is suitable for the case.

The structures of the 1D and 2D tissues are as shown in Figure 1. The pacemakers are in the central region, composed of pacing cells which are transformed from the ventricular myocytes by depressing *I*_K1_ to 0.05 nS/pF. The radius of the pacemaker is increased progressively until the pulses generated automatically from the pacemaker propagate successfully to the surrounding ventricular tissue.

The NPRs corresponding to the different diffusion coefficient *D* are shown in Table 9.

Next, the relationship between gap junction *G* and diffusion coefficient *D* is introduced first. The diffusion throughout a coupled tissue depends on the presence of the gap junction *G*. And *D* is also a tensor describing the conductivity of the tissue.

In the study, Equation (3) is adopted from reference [31] to describe the propagation of the electrical excitation wave in the cardiac tissue, which could be discretized as Equation (18).

(18) $$\begin{array}{c} C_{\text{m}}\frac{\partial V}{\partial t}=-\left(I_{\text{ion}}+I_{\text{stim}}\right)+D\Delta V=-\left(I_{\text{ion}}+I_{\text{stim}}\right)+\frac{D}{{\left(\text{dh}\right)}^{2}}\overset{n}{\underset{k=1}{\sum }}\left(V_{k}-V\right), \end{array}$$

where dh is the space step and is set to 0.15 mm in the simulation. What is more, *I*_stim_ is set to 0 in the paper, because the electrical excitation is generated from the pacemaker and the external stimulus is not required. As a consequence, the final discretized reaction diffusion equation adopted in the paper is Equation (19).

(19) $$\begin{array}{c} C_{\text{m}}\frac{\partial V}{\partial t}=-I_{\text{ion}}+\frac{D}{0.15^{2}}\overset{n}{\underset{k=1}{\sum }}\left(V_{k}-V\right). \end{array}$$

On the other hand, the data in Tables 1 and 2 are cited from reference [30], which are derived by Equation (20) describing the propagation of the electrical excitation.

(20) $$\begin{array}{c} C_{\text{m}}\frac{\partial V}{\partial t}=-I_{\text{ion}}+G_{\text{gap}}\overset{n}{\underset{k=1}{\sum }}\left(V_{k}-V\right). \end{array}$$

Comparing Equation (19) with Equation (20), it is found that *G*_gap_ is corresponding to *D*/0.15^2^. That is, except for a coefficient, *G*_gap_ and *D* are essentially the same. As a consequence, the data about NPR, *D*, and *i* will be suitable to evaluate the formula (8).

Based on formula (8), we perform the analogous analysis using the cftool in MATLAB and derive the quantitative relationship among the NPR (*N*(*i*, *D*)), the diffusion coefficient *D*, and the dimension *i*, described in Equation (21).

(21) $$\begin{array}{c} N\left(i,D\right)=0.5902*{\left(59.12\sqrt{D}\right)}^{i}. \end{array}$$

**Figure 10:**
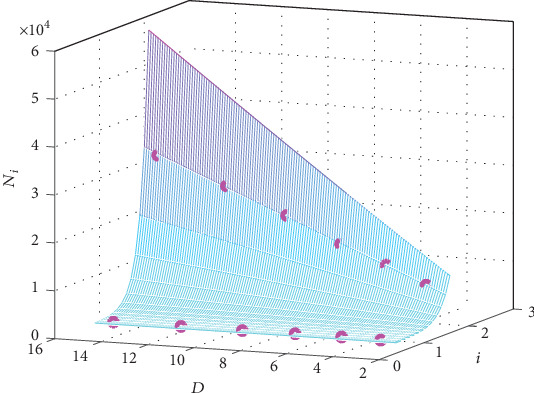
The graph of the fitting formula for the biological pacemaker. The pink circles present the original *N*_*i*_ in Table 9.

The graph of the function is shown in Figure 10 together with the 12 original values in Table 9 to intuitively evaluate the fitting of the function (21).

Figure 10 illustrates that the original values are close to the surface of the function, which suggests that the function fits the data well.

According to formula (21), when *i* = 1, the equation could be rewritten as

(22) $$\begin{array}{c} N\left(D\right)=N\left(1,D\right)=34.8926*\sqrt{D}. \end{array}$$

**Figure 11:**
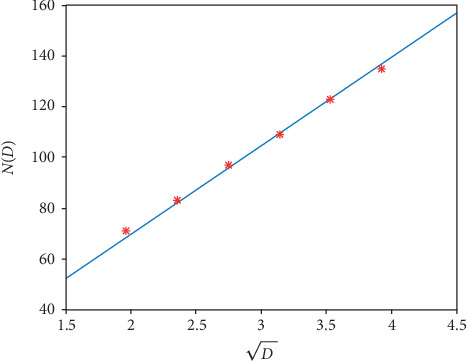
The linear graph of the function (22). The red stars are the original values in Table 9 for the 1D pacemaker.

Based on formula (22), the $\sqrt{D}‐N\left(D\right)$ graph is shown in Figure 11 together with the corresponding original values in Table 9.

And, when *i* = 2,

(23) $$\begin{array}{c} N\left(D\right)=N\left(2,D\right)=2062.8518*D. \end{array}$$

**Figure 12:**
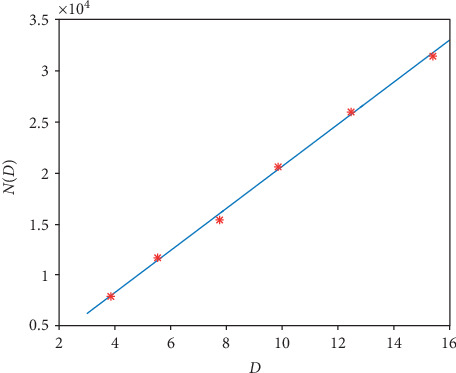
The linear graph of the function (23). The red stars are the original values in Table 9 for the 2D pacemaker.

The graph of function (23) is shown in Figure 12 together with the corresponding original values in Table 9.

The goodness of fit (*R*^2^) for formula (22) and formula (23) are 0.9998 and 0.9999, respectively, which demonstrates the fitting is accurate.

In summary, based on the relationship between NPR and *G* in references [28–30] and the relationship between NPR and *i* found in the study, together with the data from reference [30] and the simulation in the paper, the relationship among the NPR (*N*(*i*, *G*)), the dimension *i*, and the gap junction *G* (diffusion coefficient *D*) is derived and evaluated, which satisfies the two-variable function in Equation (8).

## 4. Conclusions

In the previous studies, the data are analyzed separately in three groups according to the dimensions and not treated as a whole. And only the relationship between NPR and *G* is derived for each fixed dimension *i*. As a consequence, there are diverse formulas for different dimensions. In the study, we further find the relationship between NPR and *i* for each fixed *G*. Finally, based on the two relationships and all the data treated as a whole, the relationship among the three is obtained and described in Equation (8), which shows the NPR scales with the *i* (the dimension) power of the product of the square root of the diffusion coefficient *D* (coupling or gap junction *G*) and a constant *b* which might depend on the strength of the pacing cells. And then, the accuracy of the formula is evaluated by the goodness of fit (*R*^2^), inferring that the formula fits the data well. Moreover, a 1D strand model and a 2D tissue model are developed to derive more data to verify the formula, and a positive result is received.

In conclusion, based on the data from the reference and the simulation, the formula $N\left(i,G\right)=a*{\left(b\sqrt{G}\right)}^{i}$ is proposed and validated to describe the relationship among the NPR *N*(*i*, *G*), the dimension *i*, and the diffusion coefficient *D* (coupling or gap junction *G*). And only one formula is required for all the data in the three dimensions in the study, while three formulas are needed according to the previous studies. Moreover, especially for the 3D simulation, there are always 10^6^~10^7^ cells in a volume. It will cost a lot of time only for a single simulation period. Therefore, much time is spent on finding the suitable NPR. However, according to the results in the work, formula (8) could be determined in advance based on the 1D and 2D data. And then, the NPR for the 3D simulation could be predicted by the determined formula. As a result, much time is saved.

On the other hand, more simulation and experimental data are expected to verify the formula; what is more, further investigation is necessary to probe the biological meaning of the parameters *a* and *b*.

## Figures and Tables

**Table 1:** Number of contiguous myocytes with an EAD required to trigger an excitation propagation in 1D, 2D, and 3D tissues [30].

EADs	EAD/normal	EAD/normal	EAD/RRR	EAD/RRR
	*G*_gap_ = 780 nS	*G*_gap_ = 125 nS	*G*_gap_ = 780 nS	*G*_gap_ = 125 nS
*N*_1_ (1D)	70	30	46	20
*N*_2_ (2D)	6940	1135	3217	531
*N*_3_ (3D)	696,910	50,965	229,850	17,157

**Table 2:** Number of contiguous myocytes with a DAD required to trigger an excitation propagation in 1D, 2D, and 3D tissues [30].

DADs	DAD/normal	DAD/normal	DAD/HF	DAD/HF
	*G*_gap_ = 780 nS	*G*_gap_ = 125 nS	*G*_gap_ = 780 nS	*G*_gap_ = 125 nS
*N*_1_ (1D)	80	32	22	10
*N*_2_ (2D)	7854	1256	1018	202
*N*_3_ (3D)	817,280	57,906	50,965	4189

**Table 3:** The abbreviations in Tables 1 and 2.

EAD	Early afterdepolarization
DAD	Delayed afterdepolarization
RRR	Reduced repolarization reserve
HF	Heart failure
EAD/normal	EAD-susceptible pacemaker in the central region surrounded by normal ventricular myocytes
EAD/RRR	EAD-susceptible pacemaker in the central region surrounded by ventricular myocytes with RRR changes
DAD/normal	DAD-susceptible pacemaker in the central region surrounded by normal ventricular myocytes
DAD/HF	DAD-susceptible pacemaker in the central region surrounded by ventricular myocytes with HF changes

**Table 4:** The quantitative relationship among *N*_1_, *N*_2_, and *N*_3_ for EADs.

EADs	EAD/normal	EAD/normal	EAD/RRR	EAD/RRR
	*G*_gap_ = 780 nS	*G*_gap_ = 125 nS	*G*_gap_ = 780 nS	*G*_gap_ = 125 nS
*N*_2_/*N*_1_	99.14	37.83	69.93	26.55
*N*_3_/*N*_2_	100.42	44.90	71.45	32.31
(*N*_2_/*N*_1_)/(*N*_3_/*N*_2_)	0.99	0.84	0.98	0.82

**Table 5:** The quantitative relationship among *N*_1_, *N*_2_, and *N*_3_ for DADs.

DADs	DAD/normal	DAD/normal	DAD/HF	DAD/HF
	*G*_gap_ = 780 nS	*G*_gap_ = 125 nS	*G*_gap_ = 780 nS	*G*_gap_ = 125 nS
*N*_2_/*N*_1_	98.18	39.25	46.27	20.20
*N*_3_/*N*_2_	104.06	46.10	50.06	20.74
(*N*_2_/*N*_1_)/(*N*_3_/*N*_2_)	0.94	0.85	0.92	0.97

**Table 6:** The relationship between *q* and *G*_gap_.

	EAD/normal	EAD/RRR	DAD/normal	DAD/HF
${\bar{q}}_{780}\left(G_{\text{gap}}=780\text{ nS}\right)$	99.780	70.690	101.120	48.165
${\bar{q}}_{125}\left(G_{\text{gap}}=125\text{ nS}\right)$	41.365	29.430	42.675	20.470
${\bar{q}}_{780}/{\bar{q}}_{125}$	2.412	2.402	2.370	2.353
$\sqrt{G_{780}}/\sqrt{G_{125}}$	2.50	2.50	2.50	2.50
$\left({\bar{q}}_{780}/{\bar{q}}_{125}\right)/\left(\sqrt{G_{780}}/\sqrt{G_{125}}\right)$	0.965	0.961	0.948	0.941

**Table 7:** The computing *N*(*i*, *G*) and the original values.

EAD/normal	*G*_gap_ = 780 nS, 3D	*G*_gap_ = 780 nS, 2D	*G*_gap_ = 780 nS, 1D	*G*_gap_ = 125 nS, 3D	*G*_gap_ = 125 nS, 2D	*G*_gap_ = 125 nS, 1D
*N*(original)	696,910	6940	70	50,960	1135	30
*N*(*i*, *G*)	697,437	6944	69	44,743	1113	28
*N*(*i*, *G*)/*N*	1.008	1.006	0.9878	0.8780	0.9805	0.9227

**Table 8:** The goodness of fit (*R*^2^).

	EAD/normal	EAD/RRR	DAD/normal	DAD/HF
*G*_gap_ = 125 nS	0.9964	0.9985	0.9995	0.9922
*G*_gap_ = 780 nS	1.0000	1.0000	0.9999	0.9998

**Table 9:** Number of contiguous pacing cells required to trigger the excitation propagation in the 1D and 2D tissues.

	*D* = 3.85 mm^2^/s	*D* = 5.544 mm^2^/s	*D* = 7.546 mm^2^/s	*D* = 9.856 mm^2^/s	*D* = 12.474 mm^2^/s	*D* = 15.4 mm^2^/s
*N*_1_ (1D)	71	83	97	109	123	135
*N*_2_ (2D)	7845	11,681	15,373	20,593	25,997	31,417

## Data Availability

The data supporting this research are from previously reported studies, which have been cited.
